# Age-dependent changes of gender disparities in nasopharyngeal carcinoma survival

**DOI:** 10.1186/s13293-021-00361-8

**Published:** 2021-01-30

**Authors:** Wang-Zhong Li, Shu-Hui Lv, Guo-Ying Liu, Hu Liang, Wei-Xiong Xia, Yan-Qun Xiang

**Affiliations:** 1 State Key Laboratory of Oncology in South China, Collaborative Innovation Center for Cancer Medicine, Guangdong Key Laboratory of Nasopharyngeal Carcinoma Diagnosis and Therapy, Sun Yat-Sen University Cancer Center, 651 Dongfeng Road East, Guangzhou, 510060 China; 2 Department of Nasopharyngeal Carcinoma, Sun Yat-Sen University Cancer Center, 651 Dongfeng Road East, Guangzhou, 510060 China

**Keywords:** Nasopharyngeal carcinoma, Gender disparities, Age-dependent changes, Cancer survival

## Abstract

**Background:**

The mortality of nasopharyngeal carcinoma (NPC) is usually lower in females than in males, but the underlying mechanism remains largely unknown. We sought to describe the age-dependent patterns of gender disparities in NPC survival and explore the extent to which the confounder or mediation effects could explain these differences.

**Methods:**

A total of 11,980 patients with NPC were reviewed. The effect of gender on cancer-specific survival (CSS) was assessed using Cox regression analyses. Two propensity score methods were conducted to control the confounding bias between genders. Restricted cubic spline regression was used to model the association of gender and age with mortality flexibly. Multiple mediation analysis was applied to estimate the direct or indirect effect of gender on CSS.

**Results:**

Overall, 7026 males and 2320 females were analyzed. The crude CSS was significantly higher for females than males (10-year CSS 78.4% vs 70.3%; *P* < 0.001). Similar results were observed after adjusting for confounding bias. Gender disparities in NPC-specific mortality were age-dependent, where they would increase with age until peaking at age 55–60 years and decline rapidly afterward. Subgroup analyses revealed that females’ survival advantage was observed in the 18–45 age group and was more prominent in the 46–55 age group, but vanished in the > 55 age group. Either confounder or mediation effects only accounted for approximately 20% of the gender differences.

**Conclusions:**

Gender disparities in cancer-specific mortality for patients with NPC were age-dependent. The differences mostly cannot be explained by confounder or mediation effects.

**Supplementary Information:**

The online version contains supplementary material available at 10.1186/s13293-021-00361-8.

## Introduction

Nasopharyngeal carcinoma (NPC) is characterized by a unique geographical distribution [1]. It is prevalent in certain regions, such as southern China and Southeast Asia [2]. Male patients account for the majority of NPC cases. The age-standardized incidence and mortality rates of NPC are higher in males than females. According to the Global Cancer Statistics estimates, males have a 2.75-fold higher risk of being diagnosed with NPC, while a 3.25-fold more increased chance of dying of NPC than their female counterparts [3]. The disproportionately lower mortality rates relative to incidence in females suggest a survival advantage of the female sex. However, previous studies conducted in different settings showed inconsistent results [4–15]. Female sex as an independent prognostic factor in NPC remains mostly undefined.

Many lines of evidence indicated that gender disparity in cancer survival could be partially attributed to gender-specific risk exposures [16–22]. For instance, older age at diagnosis, more advanced stage at presentation, more aggressive tumor biology, less optimal treatment, and risky behaviors like smoking and alcohol consumption in males may contribute to the survival differences. However, even after appropriate adjustment of many known risk factors, gender disparity in cancer survival is sustained. Growing evidence suggested that distinct biological determinants, such as sex hormones and sex chromosomes, could be a potential mechanism that drives females’ survival advantage [16].

In the current study, we hypothesized that the survival advantage associated with the female sex would not be constant throughout their lifecycle and might vary due to changeable gender-associated intrinsic determinants by age. Using a sizeable institutional cohort, we aimed to examine the age-dependent pattern of gender disparity in NPC-specific survival. We also evaluated the extent to which the survival difference between males and females could be explained by mediation or confounder effects.

## Patients and methods

### Participants

From January 2000 to December 2011, a total of 11,980 patients with NPC who had undergone definitive radiotherapy at Sun Yat-Sen University Cancer Center (SYSUCC) were retrospectively reviewed. Individual-level data on clinicopathological characteristics, demographic variables, treatment information, and survival outcomes were extracted from a prospectively maintained institutional database. There were 2634 patients excluded, including foreign patients (*n* = 50), patients younger than 18 years old (*n* = 802), patients without histological diagnosis (*n* = 6), patients with relapses or distant metastases (*n* = 1674), and patients with previous or synchronous malignant tumors (*n* = 102). The study was approved by the SYSUC Institutional Review Board. Patient informed consent was waived owing to the use of anonymous retrospective data. The key raw data analyzed in this study has been uploaded onto the Research Data Deposit platform (www.researchdata.org.cn) with approval RDD number RDDA2020001607.

### Study variables

Variables of interest included in analyses were gender, age, comorbidity, drinking status, smoking status, body mass index (BMI), histology, viral capsid antigen immunoglobulin A (IgA), early antigen IgA, tumor stage, node stage, clinical stage, treatment modality, radiotherapy technique, education level, employment, and marital status. We classified the disease stage using the 7th edition of American Joint Committee on Cancer TNM staging manual [23]. We categorized the histological subtypes according to the World Health Organization subtypes as type I (squamous cell carcinoma), type II (keratinizing undifferentiated carcinoma), and type III (non-keratinizing undifferentiated carcinoma). Individual education level was classified into three categories according to the International Standard Classification of Education (ISCED, 2011 version) as follows: low (ISCED 0-2: less than primary education, primary education, and lower secondary education), medium (ISCED 3–4: upper secondary education), and high (ISCED 5–6: tertiary education). Employment was classified as employed, unemployed, and retired. Marital status was grouped as married and unmarried.

### Treatment and follow-up

All study patients received definitive radiotherapy as the primary treatment with or without chemotherapy. The treatment modalities were made based on the National Comprehensive Cancer Network guidelines, institutional practice, patient preference, and treatment tolerance. Generally speaking, patients with early NPC received radical radiotherapy alone, whereas patients with locoregionally advanced NPC were treated by radiotherapy combined with platinum-based induction, concurrent, or adjuvant chemotherapy. The radiation techniques used in our institution included conventional radiotherapy and intensity-modulated radiotherapy. Details of radiation techniques we used have been reported previously [10].

After completing treatment, patients were followed every 3 months during the first 3 years, every 6 months during the next 2 years, and annually after that. Follow-up was achieved by checking the medical records of routine clinic visits or telephone calls. The primary outcome was cancer-specific survival (CSS), which was calculated from the date of diagnosis to the date of death from NPC but rather than other causes. Patients were censored at the last clinic visit or previous contact.

### Statistical analysis

Descriptive characteristics were compared using a Student’s *t* test for continuous variables and Pearson *χ*^2^ test for categorical variables. Survival curves were plotted using the Kaplan-Meier method, and survival differences were compared using the log-rank test. Cox proportional hazard regression models were used to evaluate the prognostic value of gender and other confounding factors. Given the observed gender difference in descriptive characteristics, two propensity score methods, including propensity score matching (PSM) and inverse probability of treatment weighting (IPTW), were used to reduce or eliminate the effect of unbalanced characteristics between both genders [24]. The propensity score for each patient was calculated using logistic regression. The variables such as age, comorbidity, drinking status, smoking status, BMI, histology, tumor stage, node stage, clinical stage, treatment modality, and radiotherapy technique were included in the propensity score model. The balance in baseline characteristics between male and female patients was examined using the standardized mean difference (SMD). An SMD lower than 0.1 was considered as a sign of adequate balance.

Interactions of gender and age with mortality rate were evaluated with stratified analyses and maximum likelihood tests. We used restricted cubic splines to flexibly model and visualize the association of gender and age with mortality. Estimates were adjusted for comorbidity, drinking status, smoking status, body mass index, histology, tumor stage, node stage, clinical stage, and treatment modality, and stratified for radiotherapy technique. Since most females would enter menopause after 55 years old, we selected 55 years as a reference, where the estimated HR was equal to one [25]. We compared gender differences in NPC survival in three specific age strata: 18–44 age group (premenopausal period), 45–55 age group (perimenopausal period), and > 55 age group (postmenopausal period). Likewise, two propensity score methods mentioned above were used to reduce the possible selection biases. To evaluate whether mediation effects could explain the survival difference between males and females, we performed multiple mediation analyses to quantify the direct and indirect impact of gender on NPC survival [26].

All statistical analyses were performed by using R 3.6.3 (https://www.r-project.org/). The threshold of significance was set at a two-sided *P* < 0.05.

## Results

### Patient characteristics

This study included 9346 patients with NPC diagnosed from 2000 to 2011, of whom 7026 were males and 2320 were females. Differences in patient characteristics between genders are summarized in Table 1. Female patients presented with younger age (44.8 vs 46.7 years, *P* < 0.001), less comorbidity (19.1% vs 24.3%, *P* < 0.001), lower BMI (22.2 vs 23.1, *P* < 0.001), and lower portion of smoking (1.8% vs 58.0%, *P* < 0.001) and alcohol consumption (0.9% vs 20.0%, *P* < 0.001). Male patients had higher proportions of advanced NPC (stage IV 31.0% vs 24.6%, *P* < 0.001) and high education level (22.5% vs 17.0%, *P* < 0.001) and were more likely to received radiotherapy alone (30.4% vs 28.8%, *P* = 0.047).

**Table 1:** Patient characteristics stratified by gender

Variable	Total (*N* = 9346)	Male (*N* = 7026)	Female (*N* = 2320)	*P* value
Age, mean ± SD	46.2 ± 11.2	46.7 ± 11.2	44.8 ± 11.3	< 0.001
Comorbidity				< 0.001
No	7193 (77.0%)	5317 (75.7%)	1876 (80.9%)	
Yes	2153 (23.0%)	1709 (24.3%)	444 (19.1%)	
Drinking status				< 0.001
No	7919 (84.7%)	5619 (80.0%)	2300 (99.1%)	
Yes	1427 (15.3%)	1407 (20.0%)	20 (0.9%)	
Smoking status				< 0.001
No	5227 (55.9%)	2949 (42.0%)	2278 (98.2%)	
Yes	4119 (44.1%)	4077 (58.0%)	42 (1.8%)	
BMI, mean ± SD	22.9 ± 3.24)	23.1 ± 3.19	22.2 ± 3.29	< 0.001
Histology type				0.714
I	38 (0.41%)	28 (0.40%)	10 (0.43%)	
II	494 (5.29%)	364 (5.18%)	130 (5.60%)	
III	8814 (94.3%)	6634 (94.4%)	2180 (94.0%)	
VCA IgA				0.334
0–40	1889 (20.2%)	1399 (19.9%)	490 (21.1%)	
80–320	3071 (32.9%)	2303 (32.8%)	768 (33.1%)	
> 320	4386 (46.9%)	3324 (47.3%)	1062 (45.8%)	
EA IgA				0.001
0–10	3249 (34.8%)	2371 (33.7%)	878 (37.8%)	
20–80	3951 (42.3%)	3009 (42.8%)	942 (40.6%)	
> 80	2146 (23.0%)	1646 (23.4%)	500 (21.6%)	
T category				< 0.001
T1	838 (8.97%)	653 (9.29%)	185 (7.97%)	
T2	2388 (25.6%)	1756 (25.0%)	632 (27.2%)	
T3	3930 (42.1%)	2875 (40.9%)	1055 (45.5%)	
T4	2190 (23.4%)	1742 (24.8%)	448 (19.3%)	
N category				< 0.001
N0	2078 (22.2%)	1616 (23.0%)	462 (19.9%)	
N1	3659 (39.2%)	2664 (37.9%)	995 (42.9%)	
N2	2908 (31.1%)	2188 (31.1%)	720 (31.0%)	
N3	701 (7.50%)	558 (7.94%)	143 (6.16%)	
Clinical stage				< 0.001
I	322 (3.45%)	258 (3.67%)	64 (2.76%)	
II	1755 (18.8%)	1282 (18.2%)	473 (20.4%)	
III	4518 (48.3%)	3305 (47.0%)	1213 (52.3%)	
IV	2751 (29.4%)	2181 (31.0%)	570 (24.6%)	
Treatment modality				0.047
RT alone	2805 (30.0%)	2136 (30.4%)	669 (28.8%)	
CCRT	2263 (24.2%)	1662 (23.7%)	601 (25.9%)	
IC + CCRT	4008 (42.9%)	3013 (42.9%)	995 (42.9%)	
CCRT + AC	270 (2.89%)	215 (3.06%)	55 (2.37%)	
RT technique				0.172
CRT	7042 (75.3%)	5319 (75.7%)	1723 (74.3%)	
IMRT	2304 (24.7%)	1707 (24.3%)	597 (25.7%)	
Marital status				0.137
Unmarried	429 (4.59%)	309 (4.40%)	120 (5.17%)	
Married	8917 (95.4%)	6717 (95.6%)	2200 (94.8%)	
Employment				< 0.001
Unemployed	817 (8.74%)	454 (6.46%)	363 (15.6%)	
Employed	7918 (84.7%)	6159 (87.7%)	1759 (75.8%)	
Retired	611 (6.54%)	413 (5.88%)	198 (8.53%)	
Education level				< 0.001
Low	4284 (45.8%)	3025 (43.1%)	1259 (54.3%)	
Medium	3089 (33.1%)	2423 (34.5%)	666 (28.7%)	
High	1973 (21.1%)	1578 (22.5%)	395 (17.0%)	

*Abbreviations*: *SD* standard deviation, *BMI* body mass index, *VCA* viral capsid antigen, *IgA* immunoglobulin A, *EA* early antigen, *RT* radiotherapy, *CCRT* concurrent chemo-radiotherapy, *IC* induction chemotherapy, *AC* adjuvant chemotherapy, *CRT* conventional radiotherapy, *IMRT* intensity-modulated radiotherapy

### Effect of gender on NPC-specific survival

**Fig. 1:**
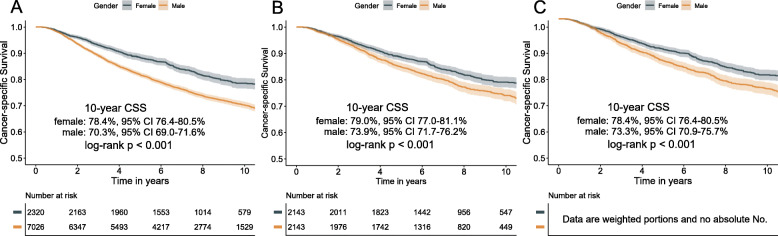
Cancer-specific survival curves stratified by gender for patients with nasopharyngeal carcinoma in the entire cohort: **a** unadjusted; **b** propensity score matching-adjusted; **c** inverse probability of treatment weighting-adjusted

With a median follow-up of 8.3 years for males and 8.37 years for females, 1795 cancer-specific deaths (25.6%) were observed in males and 419 (18.0%) in females. In crude Kaplan-Meier analysis, females had better CSS rates than that of males (5-year CSS 88.5% vs 81.9%; 10-year CSS 78.4% vs 70.3%; *P* < 0.001; Fig. 1a). In multivariate Cox regression model, after adjusting for demographic, clinical, and treatment factors, female patients still demonstrated longer CSS than male counterparts (HR 0.73, 95% CI 0.65–0.83; *P* < 0.001; Table E1). Following PSM and IPTW procedures, well balances were achieved concerning all the baseline characteristics between the two gender groups (Figure E1). PSM- and IPTW-adjusted Kaplan-Meier analyses also showed a significant difference in CSS rates between the two genders (Fig. 1b, c; both *P* < 0.001). Further multivariable analyses revealed that the gender was an independent prognostic factor both in PSM (HR 0.75, 95% CI 0.65–0.86; *P* < 0.001; Table E1) and IPTW (HR 0.75, 95% CI 0.66–0.86; *P* < 0.001; Table E1) cohorts.

### Gender-dependent association between age and cancer-specific mortality

**Fig. 2:**
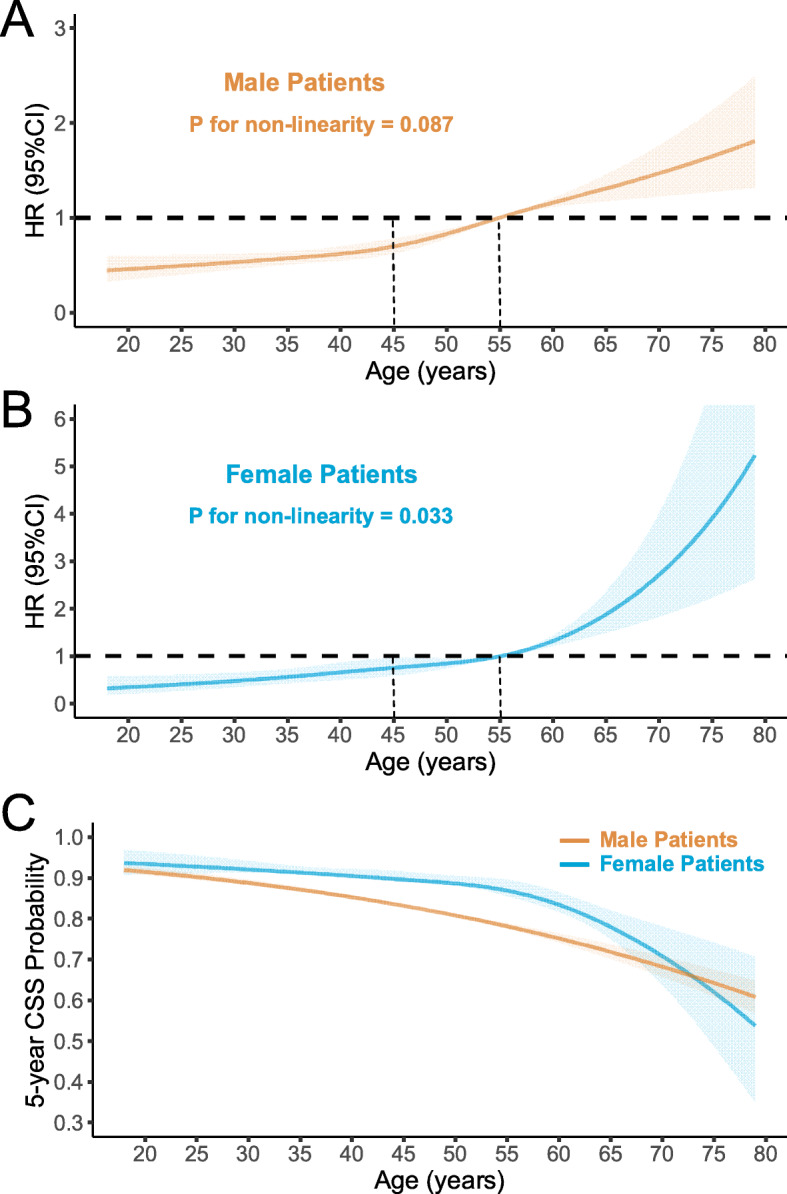
Restricted cubic splines with four knots at 35, 45, 55, and 65 were used to flexibly model and visualize the association between cancer-specific mortality risk and age. No significant non-linearity effect was observed for male patients (**a**), whereas a considerable non-linearity effect was detected in female patients (**b**). The association between the estimated 5-year cancer-specific survival (CSS) and age in male and female patients were compared (**c**)

The associations between age and cancer-specific mortality differed by gender (*P* for interaction < 0.05). Gender-dependent stratified Kaplan-Meier analyses showed that the association of age and cancer-specific mortality varied between male and female patients. Male patients had a more apparent distinction in CSS rates across different age groups than their female counterparts (Figure E2). The restricted cubic splines with four knots at 35, 45, 55, and 65 years old were used to flexibly model and visualize the association of age and cancer-specific mortality in male and female patients. In males, the risk of NPC-specific mortality increased slowly until around 45 years old and then began to increase rapidly after that (Fig. 2a, *P* for non-linearity = 0.087). In females, the risk of NPC-specific mortality increased slowly until around 55 years old and then started to increase sharply afterward (Fig. 2b, *P* for non-linearity = 0.033). As a result, gender disparity in the estimated 5- and 10-year CSS for patients with NPC increased with age until peaking at ages 55–60 years and declined rapidly afterward (Fig. 2c and Figure E3).

### Effects of gender on NPC-specific survival stratified by age strata

**Fig. 3:**
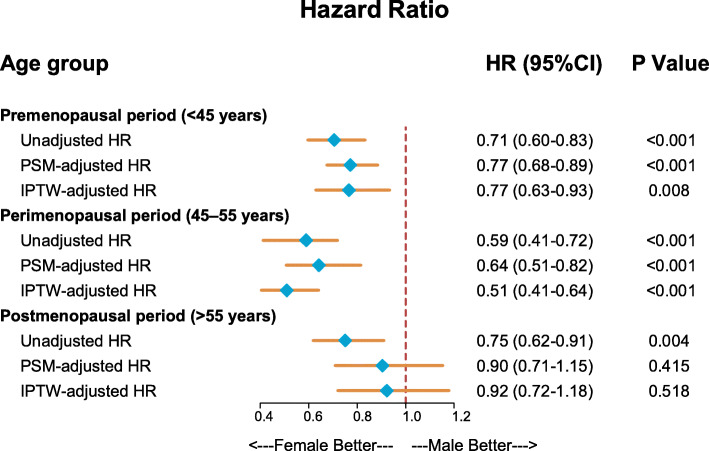
The unadjusted and adjusted effect of gender on cancer-specific survival in three different age strata (< 45 vs. 45–55 vs. > 55) were illustrated in a forest plot

The patient characteristics stratified by gender in three specific age strata were presented in Table E2. Significant gender differences were observed in most descriptive characteristics. Likewise, PSM and IPTW methods were used to reduce the observed gender differences in baseline variables. Covariate balances before and after using two propensity matching methods in premenopausal, perimenopausal, and postmenopausal age groups were shown in Figure E4–6. Excellent covariate balance was achieved across all age strata. In subgroup analyses, a lower cancer-specific mortality for females could be seen in premenopausal age group (crude HR 0.71 [0.60–0.83]; PSM-adjusted HR 0.77 [0.68–0.89]; IPTW-adjusted HR 0.77 [0.63–0.93]; all *P* < 0.001; Fig. 3). This survival advantage was more prominent in perimenopausal age group (crude HR 0.59 [0.41–0.72]; PSM-adjusted HR 0.64 [0.51–0.82]; IPTW-adjusted HR 0.51 [0.41–0.64]; all *P* < 0.001; Fig. 3). However, the significant survival advantage of the female gender vanished in the postmenopausal age group after controlling for confounding factors (crude HR 0.75 [0.62–0.91], *P* = 0.004; PSM-adjusted HR 0.90 [0.71–1.15], *P* = 0.415; IPTW-adjusted HR 0.92 [0.72–1.18], *P* = 0.518; Fig. 3).

### Direct and indirect effect of gender on NPC survival

**Fig. 4:**
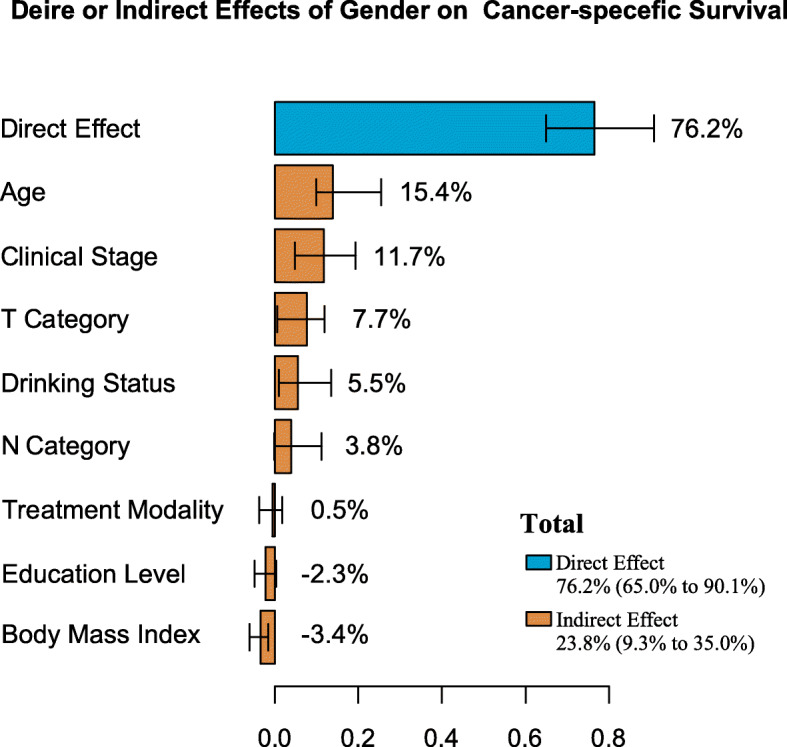
The estimation of direct and indirect effects contributing to the gender disparity in nasopharyngeal carcinoma survival was quantified using multiple mediation analyses and visualized using a bar plot with confidence intervals.

The estimated direct and indirect effects explaining gender disparities are presented in Fig. 4. Multiple mediation analyses indicated that the estimated direct effect that cannot be explained by mediating factors is 76.2% (95% CI 65.0 to 90.1%, *P* < 0.05). The proportion of indirect effect was 23.8% (95% CI 9.3 to 35.0%, *P* < 0.05). Several mediators contributing to the reduced NPC mortality among females included age at diagnosis (15.4%), drinking status (5.5%), clinical stage (11.7%), T category (7.7%), N category (3.8%), and BMI (− 3.4%).

## Discussion

To our knowledge, this the first study to investigate the age-dependent pattern of gender disparity in NPC survival using the most extensive available cohort from the endemic region. We found a significant survival advantage of females over male counterparts. Our findings agreed with several studies examining the effect of gender on NPC survival in one way or another [9–15]. Nevertheless, some methodological limitations within previous studies should be noted. A major defect of most studies is that gender disparity is described with the assumption that it would be constant throughout the lifecycle. Other concerns include insufficient follow-up duration, underlying selection bias, limited sample size, and inadequate confounding adjustment. These problems would increase the complexity of describing patterns of gender disparities due to variation in study settings, source of populations, and analytical strategy.

In the absence of convincing evidence, we investigated the effect of gender on NPC’s survival using a large sample with long-term follow-up and detailed patient characteristics. Two propensity score methods were applied to minimize potential confounding and selection bias. We also examined effect modification by age and estimated the age-dependent effects of gender on NPC-specific mortality. In the current study, gender was an independent prognostic factor. Both multivariate Cox analyses and multiple mediation analyses revealed that confounder or mediation effects only accounted for around 20% of the gender disparity in NPC survival, leaving about 80% unexplained effect that was likely determined by intrinsic factors. Furthermore, we also observed that the associations between gender and NPC mortality significantly interacted with age. Therefore, patients’ age distribution in a study will influence gender disparity in a particular population’s source.

In this study, associations between age and NPC mortality increased with age and were more potent in females than in males. Males and females showed different patterns of age-dependent NPC mortality. The risk of cancer-specific mortality in males increases rapidly after 45 years old, whereas in females increases sharply after 55 years old. As a result, significant gender disparity in NPC survival was only observed in patients younger than 55 years old after adjusting for confounding factors. They were more evident in the 46–55 age group (perimenopausal period). However, such survival benefit associated with females disappeared in the > 55 age group (postmenopausal period). Our findings were similar to a previous study examining the sex difference in NPC incidence to some extent [27]. Xie and colleagues found that the male to female ratio of NPC incidence would increase with age until peaking at ages 55–59. They suggested that the age-dependent pattern of the gender disparity in the incidence of NPC might indicate an underlying protective effect of estrogen. Our study showed some inconsistent results compared with a previous study [15]. Ouyang and colleagues found that females’ significant survival advantage in NPC mortality persisted at the premenopausal period and vanished at perimenopausal and postmenopausal periods. Nevertheless, they concluded that intrinsic biologic determinants, such as hormonal influences, might be the potential explanation.

There are preconceived notions that estrogen levels are on the decline or are low during the perimenopausal period. Different from what we expect, many studies, including well designed population-based cohort study and meta-analysis, had shown that estrogen levels were significantly higher in perimenopausal women than premenopausal women [25, 28–31]. The changing pattern of estrogen levels in females is quite in line with the age-dependent gender disparity in NPC survival we described. Therefore, contributions of the protective effect of estrogen merit consideration. Our results suggested that previous studies assuming a constant gender disparity throughout the lifecycle might lead to a misleading conclusion.

The current study has some limitations. First, data on individual income, medical insurance, residential location, and race/ethnicity are lacking and not included in analyses, which could differ between gender. These socioeconomic factors were previously shown to affect NPC survival [32–34]. The potential interactions between gender and these underlying socioeconomic factors should be considered. Second, NPC is predominantly related to Epstein­Barr virus (EBV) infection, and EBV DNA is well-established as a robust prognostic marker [1]. However, such data was generally missing in NPC patients diagnosed before 2010 at our center. The absence of this risk factor had led to the inability to evaluate its potential mediating effect with gender. Third, in some NPC cases, HPV infection is also reported, ranging from 5 to 20% [35–37]. However, this virus infection is more frequently reported in cases from non-endemic areas, especially for WHO type I NPC. As our center is in the endemic regions, testing of HPV infection in NPC cases is seldomly conducted in clinical practice. However, several recent studies from non-endemic areas report that HPV infection is not a prognostic factor in NPC [35–37]. Therefore, our results might not be influenced by the missing of HPV infection data. Finally, many factors other than sex hormones and age might play a role in better female survival. For instance, gender disparity at the cellular level has been suggested to play a role in cancer onset and progression, such as sex-specific response to stressful stimuli, genetic, and epigenetic differences determining metabolic or phenotypic traits, DNA methylation patterns, and microRNAs and related functional players [16, 38, 39]. Nevertheless, it is widely accepted that sex-related hormone signaling plays a decisive role in the gender disparity on survival.

## Perspectives and significance

Briefly, female patients with NPC had superior cancer-specific survival than their male counterparts. This gender disparity appeared to be age-dependent and mostly cannot be explained by confounder or mediation effects. The protective effect of intrinsic biologic determinants might contribute to such differences. Future research should continue to assess the remaining underlying factors that might interact with gender disparity and develop treatments tailed for males to eliminate this disparity.

## Supplementary Information

**Additional file 1: Table E1.** Results of the multivariate Cox regression models in the primary, PSM-matched, and IPTW-weighted cohort. Table E2. Patient characteristics stratified by gender in premenopausal, perimenopausal, and postmenopausal age groups. Figure E1. Covariate balance before and after using propensity matching methods in the primary cohort. An absolute mean difference of less than 0.1 indicates good balance. Figure E2. Cancer-specific survival curves with log-rank test stratified by different age strata in males (A) and females (B). Figure E3. The association between the estimated 10-year cancer-specific survival (CSS) and age in male and female patients were compared. Figure E4. Covariate balance before and after using propensity matching methods in cases younger than 45 years old. An absolute mean difference of less than 0.1 indicates good balance. Figure E5. Covariate balance before and after using propensity matching methods in cases aged 45-55 years. An absolute mean difference of less than 0.1 indicates good balance. Figure E6. Covariate balance before and after using propensity matching methods in cases older than 55 years old. An absolute mean difference of less than 0.1 indicates good balance.

## Data Availability

The authenticity of this article has been validated by uploading the key raw data onto the Research Data Deposit public platform (www.researchdata.org.cn) with the approval RDD number as RDDA2020001607.
