# Supplementary material for: Age-dependent changes of gender disparities in nasopharyngeal carcinoma survival
Source: Biol Sex Differ. 2021 Jan 30;12:18. doi: 10.1186/s13293-021-00361-8 (PMC7847041; doi:10.1186/s13293-021-00361-8)
Supplement: Supplementary file 1 — Additional file 1: Table E1. Results of the multivariate Cox regression models in the primary, PSM-matched, and IPTW-weighted cohort. Table E2. Patient characteristics stratified by gender in premenopausal, perimenopausal, and postmenopausal age groups. Figure E1. Covariate balance before and after using propensity matching methods in the primary cohort. An absolute mean difference of less than 0.1 indicates good balance. Figure E2. Cancer-specific survival curves with log-rank test stratified by different age strata in males (A) and females (B). Figure E3. The association between the estimated 10-year cancer-specific survival (CSS) and age in male and female patients were compared. Figure E4. Covariate balance before and after using propensity matching methods in cases younger than 45 years old. An absolute mean difference of less than 0.1 indicates good balance. Figure E5. Covariate balance before and after using propensity matching methods in cases aged 45-55 years. An absolute mean difference of less than 0.1 indicates good balance. Figure E6. Covariate balance before and after using propensity matching methods in cases older than 55 years old. An absolute mean difference of less than 0.1 indicates good balance. [file 13293_2021_361_MOESM1_ESM.pdf]

Supplementary Materials

1. Supplementary Tables

Table E1. Results of the multivariate Cox regression models in the primary, PSM-matched, and IPTW-weighted cohort.

Table E2. Patient characteristics stratified by gender in premenopausal, perimenopausal, and postmenopausal age groups.

2. Supplementary Figures

Figure E1. Covariate balance before and after using propensity matching methods in the primary cohort. An absolute mean difference of less than 0.1 indicates good balance.

Figure E2. Cancer-specific survival curves with log-rank test stratified by different age strata in males (A) and females (B).

Figure E3. The association between the estimated 10-year cancer-specific survival (CSS) and age in male and female patients were compared.

Figure E4. Covariate balance before and after using propensity matching methods in cases younger than 45 years old. An absolute mean difference of less than 0.1 indicates good balance.

Figure E5. Covariate balance before and after using propensity matching methods in cases aged 45-55 years. An absolute mean difference of less than 0.1 indicates good balance.

Figure E6. Covariate balance before and after using propensity matching methods in cases older than 55 years old. An absolute mean difference of less than 0.1 indicates good balance.

Table E1. Results of the multivariate Cox regression models in the primary, PSM-matched, and IPTW-weighted cohort.

Variable	Primary Cohort		PSM-matched Cohort		IPTW Cohort	
	HR (95% CI)	P-value	HR (95% CI)	P-value	HR (95% CI)	P-value
Age (continuous)	1.03 [1.03, 1.03]	<0.001	1.03 [1.02, 1.04]	<0.001	1.03 [1.02, 1.04]	<0.001
Gender (female)	0.73 [0.65, 0.83]	<0.001	0.75 [0.65, 0.86]	<0.001	0.75 [0.66, 0.86]	<0.001
Comorbidity (yes)	1.04 [0.94, 1.15]	0.438	1.06 [0.89, 1.25]	0.534	1.09 [0.92, 1.28]	0.310
Smoking status (yes)	1.04 [0.94, 1.15]	0.457	0.74 [0.37, 1.49]	0.404	1.19 [0.64, 2.21]	0.579
Drinking status (yes)	1.11 [0.99, 1.25]	0.065	1.49 [0.95, 2.34]	0.085	1.48 [1.00, 2.19]	0.053
BMI (continuous)	0.96 [0.95, 0.97]	<0.001	0.95 [0.93, 0.97]	<0.001	0.95 [0.93, 0.97]	<0.001
Histology type						
Type II vs Type I	0.65 [0.39, 1.11]	0.113	0.69 [0.29, 1.64]	0.398	0.64 [0.30, 1.38]	0.254
Type III vs Type I	0.70 [0.43, 1.14]	0.152	0.75 [0.34, 1.70]	0.496	0.71 [0.35, 1.44]	0.340
VCA IgA						
80-320 vs 0-40	1.11 [0.95, 1.29]	0.203	1.11 [0.87, 1.41]	0.385	1.13 [0.89, 1.42]	0.322
>320 vs 0-40	1.05 [0.87, 1.27]	0.599	1.17 [0.87, 1.56]	0.308	1.18 [0.89, 1.56]	0.261
EA IgA						
20-80 vs 0-10	1.06 [0.92, 1.23]	0.403	0.97 [0.78, 1.21]	0.793	1.05 [0.85, 1.30]	0.648
>80 vs 0-10	1.00 [0.84, 1.20]	0.986	0.96 [0.72, 1.27]	0.754	0.97 [0.75, 1.27]	0.843
T category						
T2 vs T1	1.38 [1.09, 1.75]	0.007	1.71 [1.14, 2.56]	0.010	1.64 [1.11, 2.40]	0.012
T3 vs T1	1.53 [1.20, 1.94]	0.001	1.87 [0.99, 3.54]	0.055	1.83 [0.97, 3.48]	0.064
T4 vs T1	2.09 [1.47, 2.97]	<0.001	1.96 [1.28, 2.98]	0.002	2.03 [1.36, 3.03]	0.001
N category						
N1 vs N0	1.40 [1.23, 1.60]	<0.001	1.57 [1.27, 1.93]	<0.001	1.62 [1.32, 1.99]	<0.001
N2 vs N0	1.92 [1.67, 2.22]	<0.001	2.13 [1.68, 2.71]	<0.001	2.25 [1.79, 2.85]	<0.001
N3 vs N0	2.70 [2.05, 3.55]	<0.001	2.43 [1.45, 4.06]	0.001	2.52 [1.48, 4.31]	0.001
Clinical stage						

II vs I	1.65 [1.00, 2.72]	0.049	1.68 [0.68, 4.20]	0.263	1.47 [0.63, 3.47]	0.375
III vs I	2.17 [1.30, 3.62]	0.003	2.15 [0.85, 5.47]	0.107	1.75 [0.73, 4.19]	0.211
IV vs I	3.02 [1.70, 5.36]	<0.001	4.29 [1.49, 12.34]	0.007	3.49 [1.26, 9.65]	0.016
Treatment modality						
CCRT vs RT alone	0.76 [0.67, 0.86]	<0.001	0.64 [0.52, 0.79]	<0.001	0.65 [0.53, 0.80]	<0.001
IC+CCRT vs RT alone	0.80 [0.72, 0.90]	<0.001	0.69 [0.57, 0.84]	<0.001	0.76 [0.63, 0.90]	0.002
CCRT+AC vs RT alone	0.91 [0.71, 1.17]	0.459	0.80 [0.52, 1.25]	0.329	0.72 [0.46, 1.13]	0.150
RT technique (IMRT)	0.62 [0.55, 0.70]	<0.001	0.69 [0.57, 0.84]	<0.001	0.68 [0.57, 0.81]	<0.001
Marital status (Unmarried)	1.07 [0.86, 1.34]	0.552	1.25 [0.87, 1.80]	0.227	1.11 [0.80, 1.56]	0.529
Employment						
Employed vs Unemployed	1.14 [0.96, 1.36]	0.121	1.16 [0.89, 1.51]	0.261	1.21 [0.99, 1.49]	0.068
Retired vs Unemployed	1.06 [0.85, 1.34]	0.591	1.18 [0.84, 1.66]	0.344	1.33 [1.01, 1.76]	0.042
Education level						
Medium vs Low	0.89 [0.81, 0.98]	0.018	1.00 [0.85, 1.17]	0.994	0.95 [0.81, 1.10]	0.495
High vs Low	0.86 [0.76, 0.97]	0.015	1.00 [0.82, 1.22]	0.993	0.94 [0.77, 1.15]	0.563

Abbreviations: PSM, propensity score matching; IPTW, inverse probability of treatment weighting; HR, hazard ratio; CI, confidence interval; BMI, body mass index; RT, radiotherapy; CCRT, concurrent chemo-radiotherapy; ICT, induction chemotherapy; ACT, adjuvant chemotherapy; IMRT, intensity-modulated radiotherapy.

Table E2. Patient characteristics stratified by gender in premenopausal, perimenopausal, and postmenopausal age groups.

Variable	Premenopausal age (<45 years)			Perimenopausal age (45-55 years)			Postmenopausal age (>55 years)		
	Male (N=3199)	Female (N=1191)	P-value	Male (N=2290)	Female (N=693)	P-value	Male (N=1537)	Female (N=436)	P-value
Age, mean±SD	36.8 (5.46)	35.9 (5.82)	<0.001	49.7 (3.18)	49.6 (3.11)	0.444	62.5 (5.41)	61.7 (4.96)	0.005
Comorbidity			<0.001			0.337			0.532
No	2442 (76.3%)	1007 (84.6%)		1769 (77.2%)	548 (79.1%)		1106 (72.0%)	321 (73.6%)	
Yes	757 (23.7%)	184 (15.4%)		521 (22.8%)	145 (20.9%)		431 (28.0%)	115 (26.4%)	
Drinking status			<0.001			<0.001			<0.001
No	2676 (83.7%)	1184 (99.4%)		1760 (76.9%)	686 (99.0%)		1183 (77.0%)	430 (98.6%)	
Yes	523 (16.3%)	7 (0.59%)		530 (23.1%)	7 (1.01%)		354 (23.0%)	6 (1.38%)	
Smoking status			<0.001			<0.001			<0.001
No	1654 (51.7%)	1183 (99.3%)		774 (33.8%)	678 (97.8%)		521 (33.9%)	417 (95.6%)	
Yes	1545 (48.3%)	8 (0.67%)		1516 (66.2%)	15 (2.16%)		1016 (66.1%)	19 (4.36%)	
BMI, mean±SD	23.0 (3.27)	21.5 (3.08)	<0.001	23.3 (3.13)	23.0 (3.21)	0.013	22.9 (3.09)	23.1 (3.46)	0.246
Histology type			0.896			0.267			0.797
I	12 (0.38%)	5 (0.42%)		9 (0.39%)	3 (0.43%)		7 (0.46%)	2 (0.46%)	
II	153 (4.78%)	54 (4.53%)		120 (5.24%)	47 (6.78%)		91 (5.92%)	29 (6.65%)	
III	3034 (94.8%)	1132 (95.0%)		2161 (94.4%)	643 (92.8%)		1439 (93.6%)	405 (92.9%)	
VCA IgA			0.599			0.255			0.428
0-40	671 (21.0%)	263 (22.1%)		437 (19.1%)	144 (20.8%)		291 (18.9%)	83 (19.0%)	
80-320	1084 (33.9%)	409 (34.3%)		741 (32.4%)	237 (34.2%)		478 (31.1%)	122 (28.0%)	
>320	1444 (45.1%)	519 (43.6%)		1112 (48.6%)	312 (45.0%)		768 (50.0%)	231 (53.0%)	
EA IgA			0.036			0.061			0.358
0-10	1163 (36.4%)	482 (40.5%)		750 (32.8%)	258 (37.2%)		458 (29.8%)	138 (31.7%)	
20-80	1343 (42.0%)	477 (40.1%)		990 (43.2%)	290 (41.8%)		676 (44.0%)	175 (40.1%)	
>80	693 (21.7%)	232 (19.5%)		550 (24.0%)	145 (20.9%)		403 (26.2%)	123 (28.2%)	
T stage			0.001			<0.001			0.041
T1	317 (9.91%)	101 (8.48%)		201 (8.78%)	50 (7.22%)		135 (8.78%)	34 (7.80%)	
T2	835 (26.1%)	302 (25.4%)		571 (24.9%)	202 (29.1%)		350 (22.8%)	128 (29.4%)	
T3	1297 (40.5%)	555 (46.6%)		938 (41.0%)	329 (47.5%)		640 (41.6%)	171 (39.2%)	
T4	750 (23.4%)	233 (19.6%)		580 (25.3%)	112 (16.2%)		412 (26.8%)	103 (23.6%)	
N stage			0.019			0.002			0.189
N0	680 (21.3%)	211 (17.7%)		533 (23.3%)	145 (20.9%)		403 (26.2%)	106 (24.3%)	

N1	1231 (38.5%)	508 (42.7%)		852 (37.2%)	311 (44.9%)		581 (37.8%)	176 (40.4%)	
N2	1053 (32.9%)	394 (33.1%)		720 (31.4%)	199 (28.7%)		415 (27.0%)	127 (29.1%)	
N3	235 (7.35%)	78 (6.55%)		185 (8.08%)	38 (5.48%)		138 (8.98%)	27 (6.19%)	
Clinical Stage			0.006			<0.001			0.181
I	120 (3.75%)	30 (2.52%)		76 (3.32%)	20 (2.89%)		62 (4.03%)	14 (3.21%)	
II	605 (18.9%)	239 (20.1%)		411 (17.9%)	148 (21.4%)		266 (17.3%)	86 (19.7%)	
III	1537 (48.0%)	621 (52.1%)		1077 (47.0%)	382 (55.1%)		691 (45.0%)	210 (48.2%)	
IV	937 (29.3%)	301 (25.3%)		726 (31.7%)	143 (20.6%)		518 (33.7%)	126 (28.9%)	
Treatment modality			0.231			0.109			0.093
RT alone	870 (27.2%)	288 (24.2%)		652 (28.5%)	211 (30.4%)		614 (39.9%)	170 (39.0%)	
CCRT	803 (25.1%)	317 (26.6%)		541 (23.6%)	181 (26.1%)		318 (20.7%)	103 (23.6%)	
IC+CCRT	1418 (44.3%)	547 (45.9%)		1027 (44.8%)	288 (41.6%)		568 (37.0%)	160 (36.7%)	
CCRT+AC	108 (3.38%)	39 (3.27%)		70 (3.06%)	13 (1.88%)		37 (2.41%)	3 (0.69%)	
RT technique			0.363			0.567			0.711
2DRT	2374 (74.2%)	867 (72.8%)		1735 (75.8%)	517 (74.6%)		1210 (78.7%)	339 (77.8%)	
IMRT	825 (25.8%)	324 (27.2%)		555 (24.2%)	176 (25.4%)		327 (21.3%)	97 (22.2%)	
Marital status			0.057			0.681			0.418
Unmarried	199 (6.22%)	94 (7.89%)		65 (2.84%)	17 (2.45%)		45 (2.93%)	9 (2.06%)	
Married	3000 (93.8%)	1097 (92.1%)		2225 (97.2%)	676 (97.5%)		1492 (97.1%)	427 (97.9%)	
Employment			<0.001			<0.001			<0.001
Unemployed	202 (6.31%)	179 (15.0%)		143 (6.24%)	114 (16.5%)		109 (7.09%)	70 (16.1%)	
Employed	2993 (93.6%)	1006 (84.5%)		2116 (92.4%)	520 (75.0%)		1050 (68.3%)	233 (53.4%)	
Retired	4 (0.13%)	6 (0.50%)		31 (1.35%)	59 (8.51%)		378 (24.6%)	133 (30.5%)	
Education level			<0.001			<0.001			<0.001
Low	1171 (36.6%)	535 (44.9%)		1057 (46.2%)	413 (59.6%)		797 (51.9%)	311 (71.3%)	
Medium	1157 (36.2%)	381 (32.0%)		800 (34.9%)	194 (28.0%)		466 (30.3%)	91 (20.9%)	
High	871 (27.2%)	275 (23.1%)		433 (18.9%)	86 (12.4%)		274 (17.8%)	34 (7.80%)	

Abbreviations: SD, standard deviation; BMI, body mass index; RT, radiotherapy; CCRT, concurrent chemo-radiotherapy; IC, induction chemotherapy; AC, adjuvant chemotherapy; 2DRT, two-dimensional radiotherapy; IMRT, intensity-modulated radiotherapy.

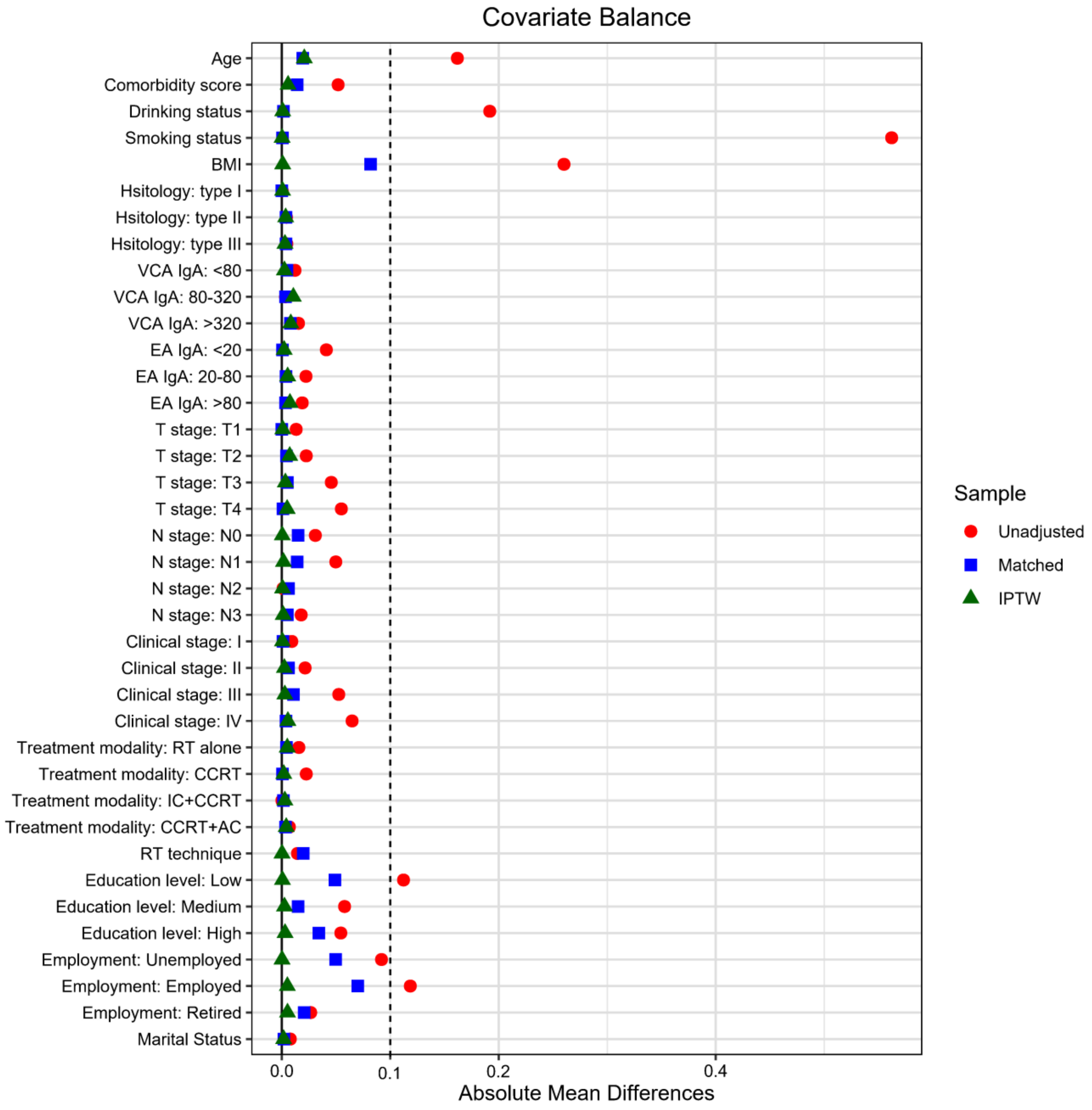

Figure E1. Covariate balance before and after using propensity matching methods in the primary cohort. An absolute mean difference of less than 0.1 indicates good balance.

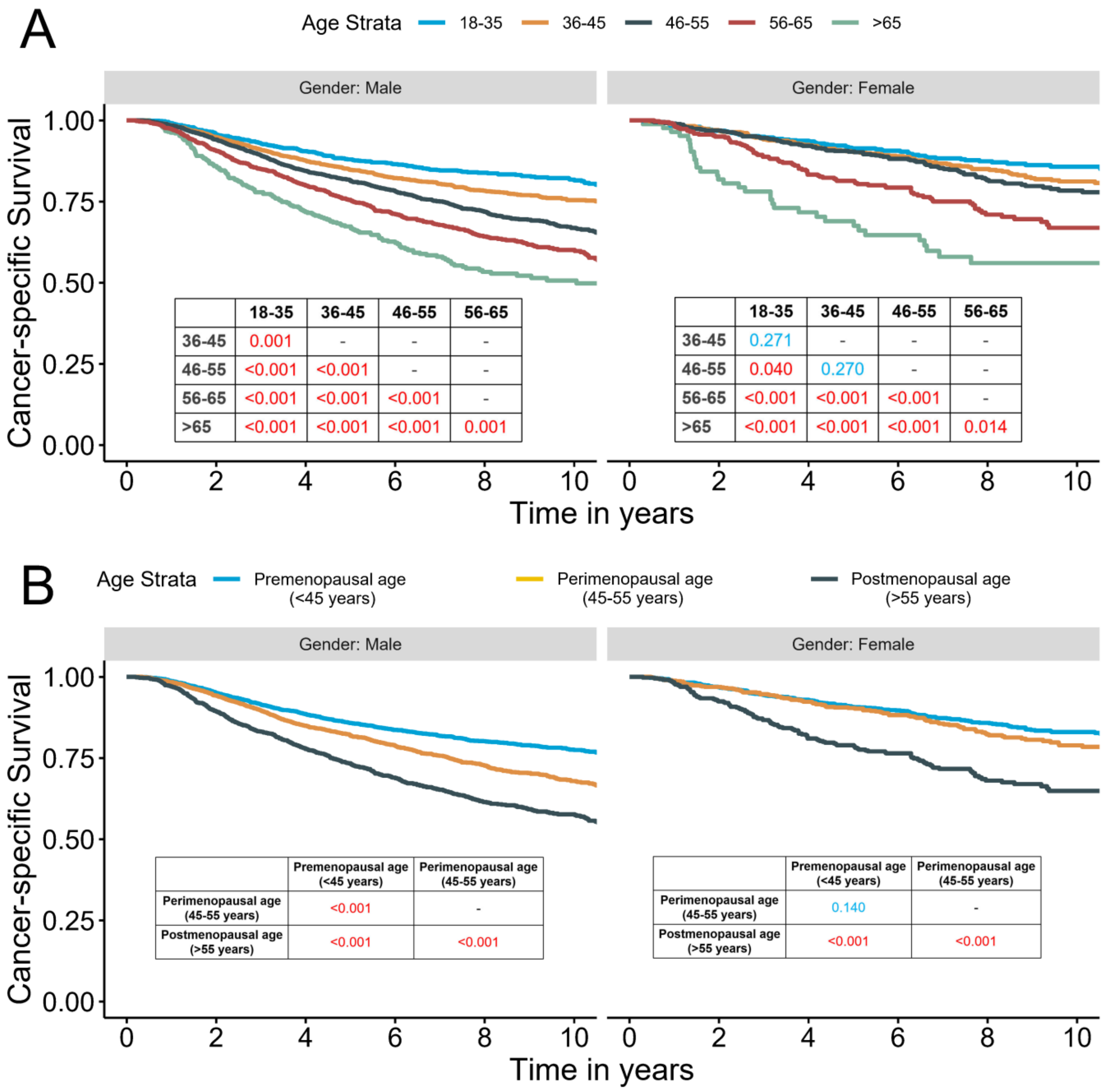

Figure E2. Cancer-specific survival curves with log-rank test stratified by different age strata in males (A) and females (B).

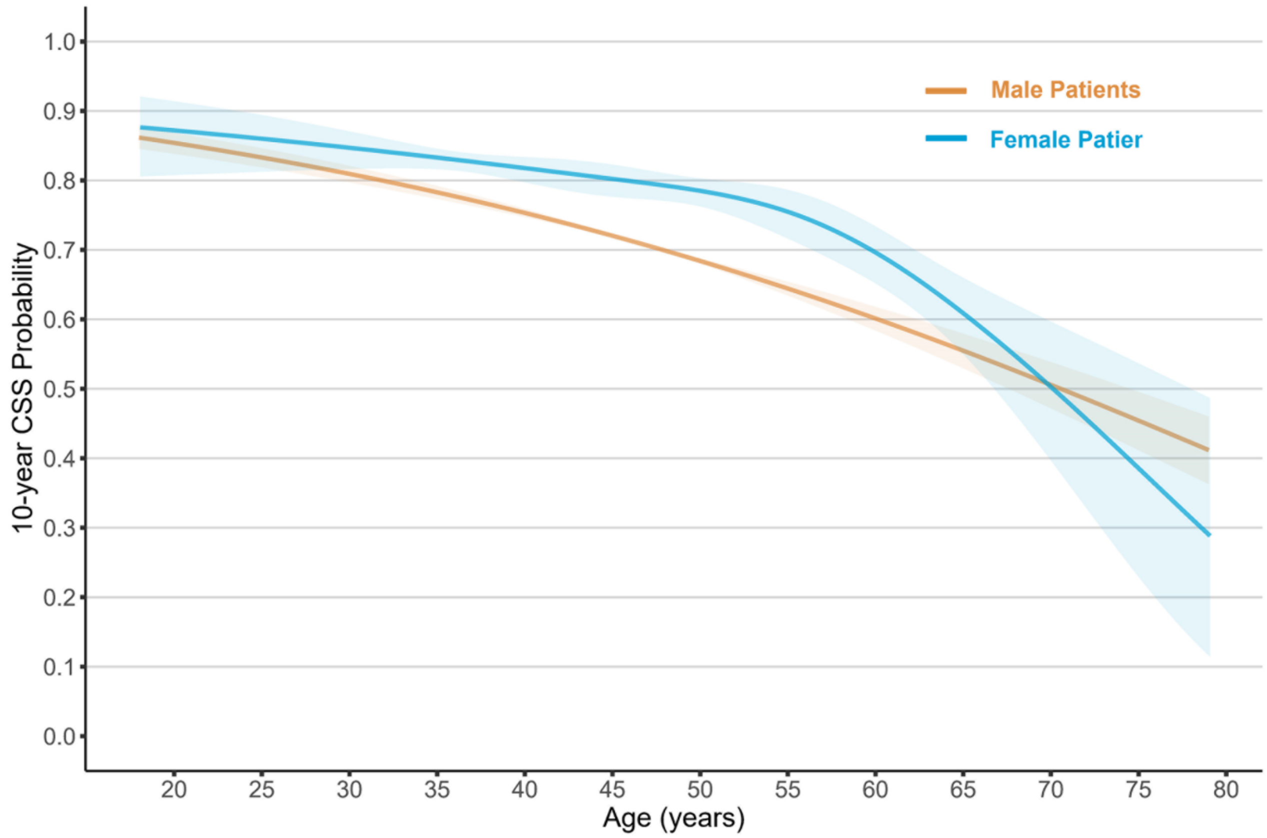

Figure E3. The association between the estimated 10-year cancer-specific survival (CSS) and age in male and female patients were compared.

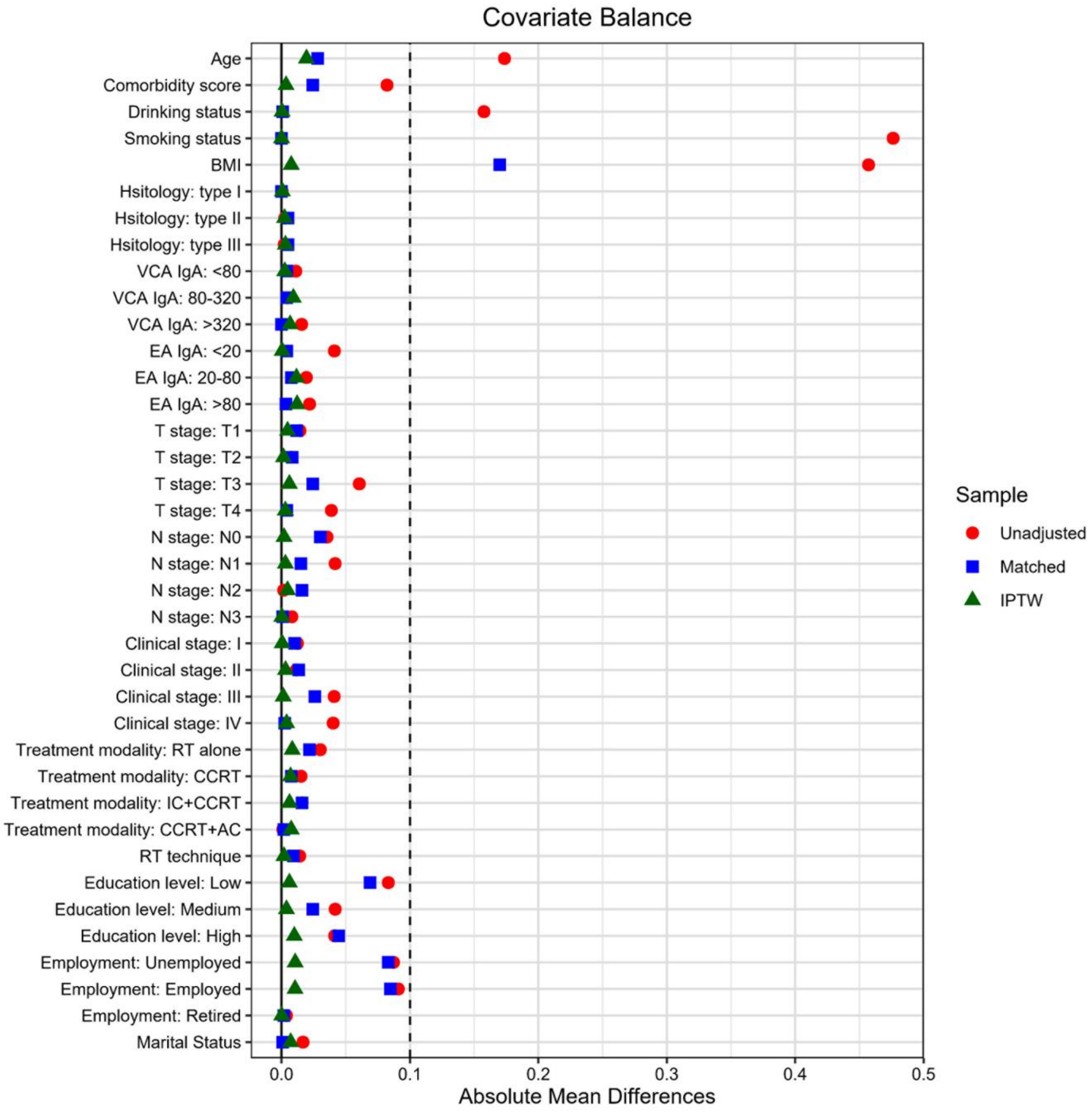

Figure E4. Covariate balance before and after using propensity matching methods in cases younger than 45 years old. An absolute mean difference of less than 0.1 indicates good balance.

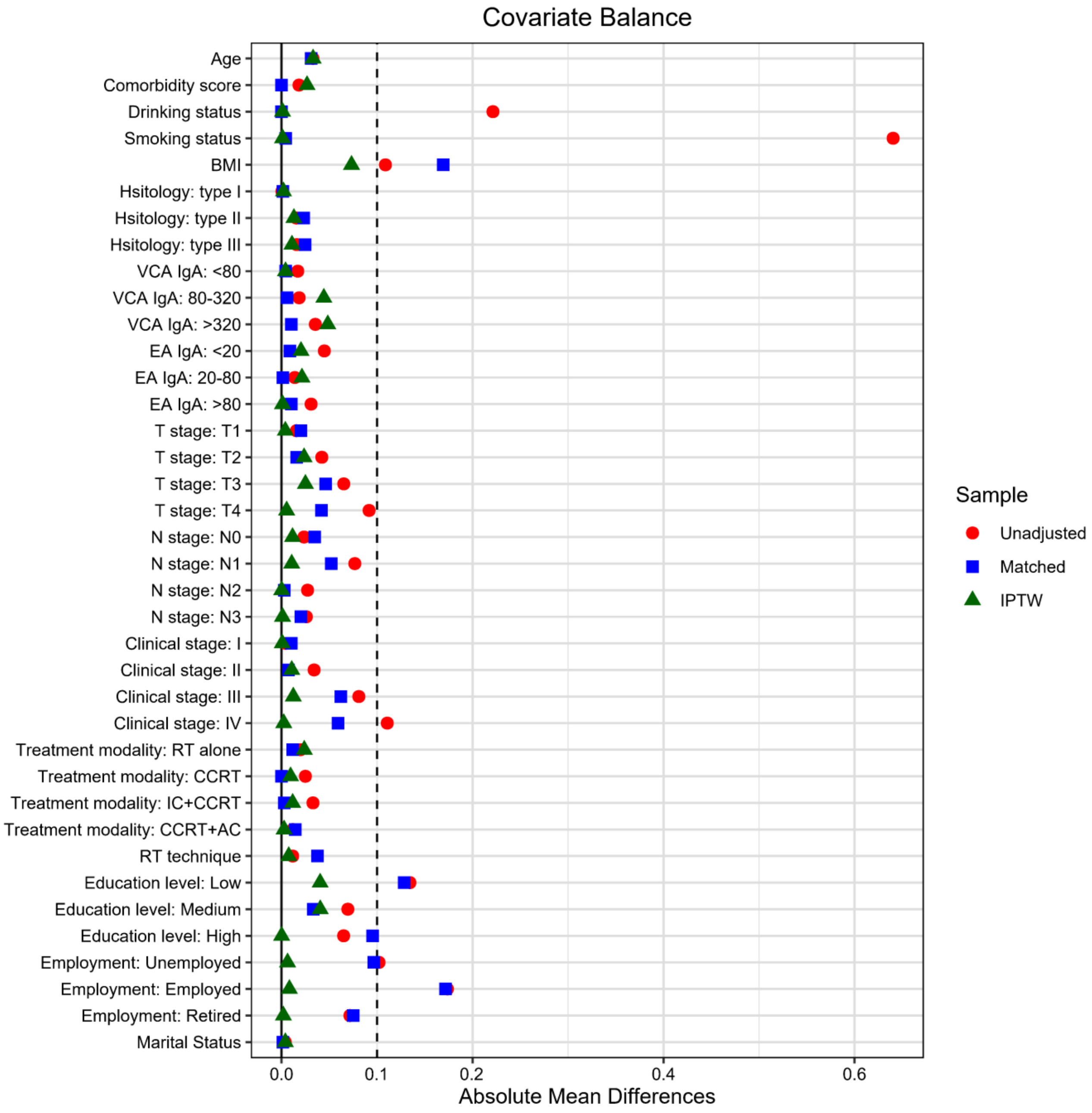

Figure E5. Covariate balance before and after using propensity matching methods in cases aged 45-55 years. An absolute mean difference of less than 0.1 indicates good balance.

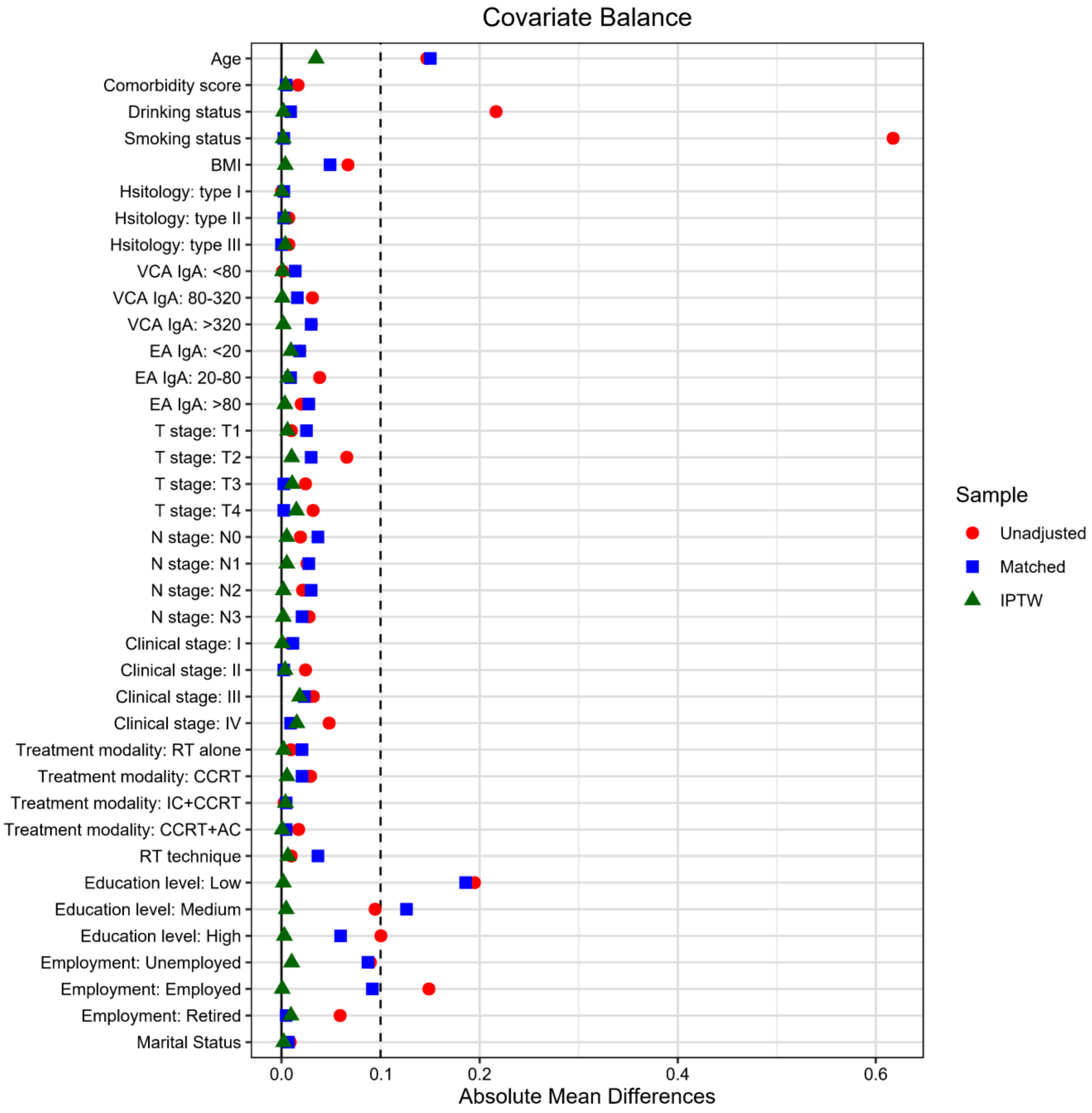

Figure E6. Covariate balance before and after using propensity matching methods in cases older than 55 years old. An absolute mean difference of less than 0.1 indicates good balance.
